# Sleep and psychiatric abnormalities in Gullian Barré Syndrome

**DOI:** 10.1186/s41983-018-0007-1

**Published:** 2018-04-25

**Authors:** Wafik Said Bahnasy, Yasser Abo Elfotoh El-Heneedy, Ahmed Mohamed El-Shamy, Marwa Yassin Badr, Reham Ahmed Amer, Ibrahim Salah Eldeen Ibrahim

**Affiliations:** 1 Department of Neuropsychiatry, Faculty of Medicine, Tanta University, Tanta, 31527 Egypt; 2 Psychiatry Unit, Department of Neuropsychiatry, Faculty of Medicine, Tanta University, Tanta, Egypt; 3 Chest Diseases Department, Faculty of Medicine, Tanta University, Tanta, Egypt

**Keywords:** Guillain Barré Syndrome, Multiple sleep latency test, Polysomnography, Hamilton anxiety scale, MADRS

## Abstract

**Background:**

The sensori-motor manifestations of Guillain Barré Syndrome (GBS) are usually severe enough to mask the psychiatric and sleep problems which are in need for more attention for better functional outcome.

**Methods:**

This study was performed on 20 GBS patients and 10 healthy controls. Patients were evaluated initially before immunotherapy using the Overall Disability Sum Score (ODSS), Neuropathy Pain Scale (NPS), Hamilton Anxiety Scale (HAS), Montgomery–Åsberg Depression Rating Scale (MADRS) and one-night polysomnography (PSG) followed by the multiple sleep latency test (MSLT) to evaluate the mean sleep latencies. Reevaluation was done using the same parameters 1 month after completing immunotherapy.

**Results:**

The study showed significant increase in HAS in GBS patients which were positively correlated with the degree of motor disability. The mean sleep latencies of MSLT were significantly shortened and PSG showed shortening of the total sleep time, sleep efficiency, lowest O_2_ saturation and pulse transit time with increased wake after sleep onset, sleep stage transition index, apnea hypopnea index, desaturation index, arousal index, snore index and periodic limb movement index. One month after immunotherapy, the anxiety symptoms and sleep abnormalities showed non-significant improvements which were not correlated with the improvements in the sensori-motor manifestations.

**Conclusions:**

GBS patients usually have sleep and psychiatric abnormalities which may take longer time to improve than the sensori-motor manifestations. So, they need more attention in the management protocol for early patients’ independence and return to usual daily activities.

## Background

Guillain Barré Syndrome (GBS) is an acute monophasic poly-radiculo-neuritis manifested by motor deficits, diminished reflexes, sensory dysfunction, and sometimes cranial nerve affection (Wakerley et al. 2014). The annual incidence of GBS is about 1–2 per 100,000, 30% of patients may need assisted ventilation, and 2–5% die of complications. Recovery from GBS usually takes long time with most of patients experience good motor outcomes and return to the usual daily activities in up to 3 years from the onset with 20% still have residual disabilities (Meena et al. 2011; Alshekhlee et al. 2008). Gullian Barré Syndrome is predominantly demyelinating in Europe and the USA and predominantly axonal in Asia, North Africa, and South America (Willison et al. 2016; van den Berg et al. 2014).

Guillain Barré Syndrome patients may experience psychiatric manifestations in the form of anxiety and depressive symptoms which contribute to their slow recovery (Darweesh et al. 2014). They also experience sleep problems in the form of REM behavior disorders and sleep apneas due to increased upper airway resistance and weakness of the respiratory muscles and centrally due to respiratory chemoreceptors hypo-responsiveness (Peralta et al. 2006).

### Aim of the work

Was to assess sleep and psychiatric abnormalities in GBS patients, their relations to the severity of sensori-motor manifestations, and the possibility of their spontaneous recovery after GBS immunotherapy.

## Methods

The present study was conducted on 20 adult GBS patients fulfilling the level 1 or 2 diagnostic certainty of The Brighton Criteria (Goodfellow and Wilson 2016) attending The Neuropsychiatry Department, Tanta University Hospitals, in the period from 1st of April to the end of December 2016. Ten healthy control subjects matching the patient’s age, sex, and body mass index (BMI) were also included. Six patients needed cerebrospinal fluid examination, who showed the cyto-albuminous dissociation needed for diagnostic certainty. Neurophysiology was done to all patients 2–3 weeks after the onset of symptoms to confirm the diagnosis and fulfill The Brighton Criteria. The neurophysiology included bilateral motor conduction studies and F-wave latencies for each of median and ulnar nerves. Bilateral motor conduction studies and H-wave latencies for common peroneal and posterior tibial nerves. At the same time, bilateral sensory conduction studies for each of median, ulnar, and sural nerves were done (sural sparing if present is confirmatory for GBS).

Exclusion criteria included GBS patients in impending need of assisted ventilation who spirometer forced vital capacity less than 20 ml/kg, patients with chronic respiratory or cardiac problems, chronic pain, BMI≥ 28 kg/m^2^, advanced metabolic or endocrinal disorders, drugs affecting sleep intake, heavy smokers or history of psychiatric disorders.

The study protocol was approved by The Research Ethics Committee and Quality Assurance Unit, Faculty of Medicine, Tanta University. Participation was voluntary, all participants received detailed information concerning the aims and possible risks of sharing in this research work, and an informed consent was obtained from all prior to the commencement in the study.

Patients were submitted to GBS management protocol including 5–7 sessions of plasmapheresis (200–250 mL/kg of plasma was exchanged over 2 weeks) or intravenous immunoglobulin (0.4 g/kg/dose for 5 doses in 5 consecutive days).

Patients were evaluated initially at the time of hospital admission and before immunotherapy using The Overall Disability Sum Score (ODSS) (Merkies et al. 2002) and The Neuropathy Pain Scale (NPS) (Jensen et al. 2006) to assess the degree of motor and sensory manifestations respectively. Psychiatric assessment was done using Hamilton Anxiety Scale (HAS) and Montgomery–Åsberg Depression Rating Scale (MADRS) for anxiety and depressive symptoms severities (Maier et al. 1988; Williams and Kobak 2008).

All subjects were submitted to one-night polysomnogram (PSG) followed in the next day by multiple sleep latency test (MSLT) to assess the mean sleep latencies (objective parameter measuring excessive daytime sleepiness). PSG and MSLT were performed by a Somon Medics Gmbh (Am SonnenstuhL63, D-97236 Rander Sacker, Germany, Type: SOMNO screen™plus, SN: 4259, kw45: 2014). The PSG included EEG channels montages (O1/A2, C3/A2, C4/A1 and O2/A1), electrooculography (LOC-A1/A2 and ROC-A1/A2), surface tibial and submental EMG, and modified V2 lead ECG. Thermal airflow sensors (thermistor) were used for nasal and oral signals, and microphone was applied for tracheal sounds assessment. Chest and abdominal efforts were measured by dual thoracoabdominal RIP (respiratory inductance plethysmography) belts. PSG parameters were scored according to The American Academy of Sleep Medicine Scoring Manual, 2012 (Grigg-Damberger 2012).

One month after completing immunotherapy, patients were reassessed using the same parameters (HAS and MADRS, PSG and MSLT) to evaluate the degree of improvements in each and correlate them with the degrees of sensori-motor changes (ODSS and NPS).

Statistical analysis was conducted using the ANOVA and Tukey’s tests. Correlation analysis was performed using Pearson’s correlation test. *P* value less than 0.05 was considered statistically significant.

## Results

The study included 20 GBS patients aged 39.05 ± 11.3 years, 13 males (65%) and 7 females (35%) with BMI 24.9 ± 2.4 kg/m^2^. Ten (50%) patients had history of upper respiratory tract infection, 4 (20%) had gastroenteritis, and 6 (30%) could not decide. The patients were admitted 12.8 ± 2.9 days following symptoms onset, and this relatively delayed admission in some patients was due to initial faulty diagnosis by family doctors and false impression of possible spontaneous recovery during early mild initial symptoms. Eighteen patients (90%) underwent plasmapheresis, and 2 (10%) received intravenous immunoglobulin, and the mean time lapses between admission and the first session of immunotherapy were 3.1 ± 1.1 days till immunotherapy became ready during which initial assessments were done.

In initial assessments, all patients had bilateral symmetrical weakness both proximal and distal, absent or diminished deep reflexes mainly the ankle jerk; 5 (25%) patients had equivocal planter response; 4 (20%) had unilateral facial palsy; 3 (15%) had facial diplegia; and 3 (15%) had bulbar symptoms. Neurophysiological examination revealed that 15 (75%) patients had predominantly demyelinating, 2 (10%) patients had predominantly axonal neuropathy, and 3 (15%) patients had normal neurophysiology.

The ODSS and NPS were significantly higher in GBS patients compared to healthy control subjects (5.9 ± 0.8 and 17.4 ± 8.3 versus 0.7 ± 0.8 and 9 ± 3.6 with *p* value < 0.0001 and 0.004 respectively). The HAS and MADRS were also significantly higher in GBS patients than healthy control subjects (25.9 ± 5.7 and 18.9 ± 10 versus 7.7 ± 2.2 and 7.4 ± 5.1 with *p* value < 0.0001 and 0.002 respectively) (Table 1). The HAS and MADRS showed non-significant correlation with the rate of initial disease progression measured by the onset-admission duration (*p*-values 0.439 and 0.965 respectively).

**Table 1:** Comparison between Gullian Barré patients and control subjects at initial assessment

	Patients		Control	ANOVA	
		Mean ± SD	Mean ± SD	*f* value	*p* value
Overall Disability Sum Score	Early	5.95 ± 0.82	0.7 0 ± 0.82	156.47	> 0.0001*
	Late	4.15 ± 0.67			> 0.0001*
Neuropathy Pain Scale	Early	17.35 ± 8.28	9.0 ± 3.59	10.09	0.004*
	Late	9.15 ± 5.02			0.998
Hamilton Anxiety Scale	Early	25.9 ± 5.7	7.7 ± 2.16	22.5	> 0.0001*
	Late	21.15 ± 5.54			> 0.0001*
MADRS	Early	18.9 ± 9.96	7.4 ± 5.08	6.74	0.002*
	Late	15.55 ± 7.12			0.033*
Mean sleep latency of MSLT (min)	Early	11.79 ± 3.97	16.64 ± 2.2	6.79	0.002*
	Late	14.23 ± 3.45			0.185
Total sleep time (hours)	Early	5.28 ± 0.43	7.29 ± 0.33	70.49	> 0.0001*
	Late	5.64 ± 0.49			> 0.0001*
Wake after sleep onset (hours)	Early	2.91 ± 0.588	0.85 ± 0.34	64.66	> 0.0001*
	Late	2.53 ± 0.41			> 0.0001*
Sleep efficiency (%)	Early	62.56 ± 5.93	86.38 ± 4.22	74.44	> 0.0001*
	Late	67.53 ± 4.57			> 0.0001*
SSTI (No of sleep stages/h)	Early	21.9 ± 6.51	8.6 ± 1.6	24.5	> 0.0001*
	Late	19.74 ± 4.36			> 0.0001*
Apnea hypopnea index (/hour)	Early	19.11 ± 7.75	5.67 ± 5.46	16.64	> 0.0001*
	Late	18.2 ± 5.15			> 0.0001*
Pulse Transit Time (msec)	Early	184.6 ± 23.2	241.4 ± 13.2	13.6	> 0.0001*
	Late	232.3 ± 12.3			> 0.0001*
Arousal index (/hour)	Early	37.6 5 ± 12.69	5.5 ± 2.63	33.64	> 0.0001*
	Late	35.4 ± 10.98			> 0.0001*
Snore Index (/hour)	Early	299.3 ± 142.25	29.1 ± 21.89	22.53	> 0.0001*
	Late	234.45 ± 81.66			> 0.0001*
Periodic limb movements index (/hour)	Early	46.35 ± 22.61	9.9 ± 3.66	13.29	> 0.0001*
	Late	41.0 ± 18.92			> 0.0001*

*Early* after admission and before immunotherapy, *Late* 1 month after immunotherapy, *MADRS* Montgomery–Åsberg Depression Rating Scale, *MSLT* multiple sleep latency test, *SSTI* sleep stage transition index

*Significant

**Fig. 1:**
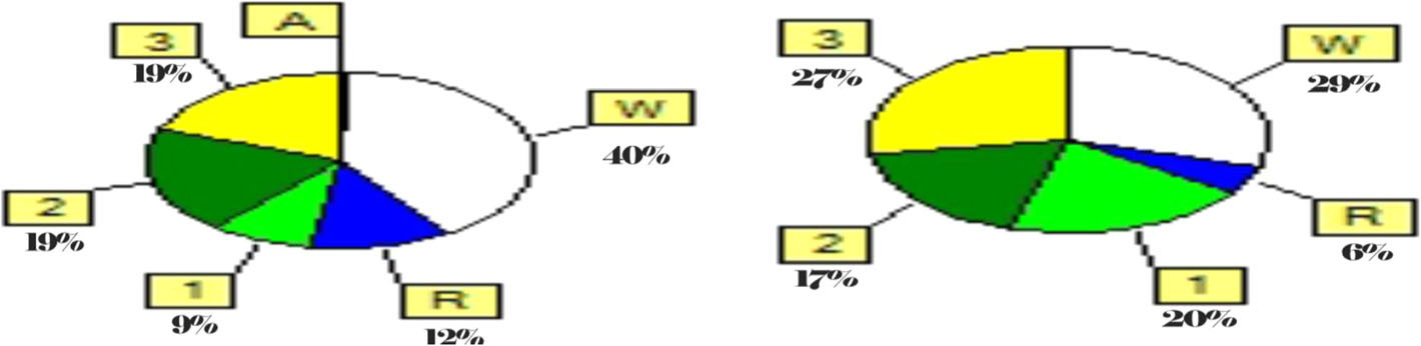
Sleep stages from total sleep time (TST) in a Gullian Barré Patient early after hospital admission (left) with marked prolongation of waking after sleep onset (W) and shortening of each N2% and REM% of TST. One month after immunotherapy (right), the same patient showed mild improvement of sleep architecture but still not reaching the normal values

The study showed significant decrease in the mean sleep latencies of the MSLT in GBS patients compared to control (11.8 ± 4 versus 16.6 ± 2.2 with *p* value 0.002). Polysomnography showed significant reduction in each of total sleep time (TST), sleep efficiency (SE), lowest O_2_ saturation, and pulse transit time (PTT) in GBS compared to healthy control subjects (5.3 ± 0.4, 62.6 ± 5.9, 86.5 ± 2.9, and 184.6 ± 23.2 versus 7.3 ± 0.3, 86.4 ± 4.2, 92.5 ± 2.5, and 241.4 ± 13.2 respectively with *p* value < 0.0001 for each). There were significant increases in wake after sleep onset (WASO), sleep stage transition index (SSTI), apnea hypopnea index (AHI), and desaturation index (DI) in GBS patients compared to healthy control subjects (2.91 ± 0.59, 21.9 ± 6.5, 19.1 ± 7.8, and 17.4 ± 7.3 versus 0.9 ± 0.3, 8.6 ± 1.6, 5.7 ± 5.5, and 6.2 ± 5.04 respectively with *p* value < 0.0001 for each). Sleep architecture of GBS patients showed prolonged WASO on the expense of shortened N2% and REM% of TST with nearly normal N1% of TST and relatively prolonged N3% of TST (19.6 ± 6.3%, 9.3 ± 3.8%, 8.1 ± 2.5%, and 24.3.3 ± 3.2% respectively) (Fig. 1).

**Fig. 2:**
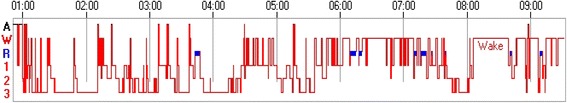
Hypnogram of a Gullian Barré patient showing increased waking after sleep onset and sleep stage transition index with shortened rapid eye movement sleep (R) and N2 non-rapid eye movement sleep (2)

There was also significant increase in each of arousal index (AI), snore index (SI), and periodic limb movement index (PLMI) in GBS patients than control (37.7 ± 12.7, 299.3 ± 142.3, and 46.4 ± 22.6 versus 5.5 ± 2.6, 29.1 ± 21.9, and 9.9 ± 3.7 respectively with *p* value < 0.0001 for each) (Table 1) (Fig. 2). Sleep and REM latencies showed non-significant difference between GBS patients and healthy control subjects. The studied GBS patients had no correlation between the evaluated PSG parameters at the time of admission and severities of motor disabilities as measured by the ODSS (except SE which showed negative correlation with ODSS) (Table 2). The PSG abnormalities are more severe in those having cranial nerve affection.

**Table 2:** Correlations of the Overall Disability Sum Score and Hamilton Anxiety Scale with other studied parameters in Gullian Barré patients at initial assessment

	Early ODSS		Hamilton Anxiety Scale	
	r	p	r	p
Neuropathic Pain Scale	0.31	0.18	0.52	0.01*
Hamilton Anxiety Scale	0.50	0.024*	–	–
Montgomery–Åsberg Depression Rating Scale	− 0.03	0.87	0.44	0.05*
Mean sleep latency of MSLT (min)	0.3	− 0.15	− 0.75	0.0001*
Total sleep time (hours)	0.40	0.07	− 0.37	0.1006
Wake after sleep onset (hours)	0.68	0.001*	0.97	< 0.0001*
Sleep efficiency (%)	− 0.69	0.0007*	− 0.89	< 0.0001*
Sleep stage transition index (/hour)	− 0.15	0.51	0.22	0.34
Apnea hypopnea index (/hour)	0.44	0.046*	0.84	< 0.0001*
Arousal index (/hour)	− 0.22	0.33	0.02	0.91
Snore Index (/hour)	0.55	0.0117*	− 0.34	0.13
Periodic limb movements index (/hour)	0.17	0.45	− 0.01	0.93

*Early ODSS* Overall Disability Sum Score on admission, *MSLT* multiple sleep latency test

*Significant

The study showed that the initial ODSS was positively correlated with the HAS, WASO, and SI and negatively correlated with SE and had no correlations with MADRS, MSLT, and other PSG parameters (Table 2). At the same time, the initial HAS measured at the time of admission was positively correlated with NPS, MADRS, WASO, AHI, and DI and negatively correlated with MSLT and SE, and had no correlation with TST, SSTI, AI, SI, and PLMI (Table 2).

The study showed that comparing GBS patients 1 month after immunotherapy with the initially evaluated healthy control subjects’ data revealed non-significant difference regarding NPS and mean latencies of MSLT with *p* value 0.99 and 0.185 respectively. Each of ODSS, HAS, and MADRS showed significant increase in GBS patients than control with *p* values < 0.0001, < 0.0001, and 0.033 respectively. At the same time, PSG showed significant reduction in each of TST, SE, and lowest O_2_ saturation in GBS compared to healthy control subjects (*p* values < 0.0001 for each). Wake after sleep onset, SSTI, AHI, DI, AI, SI, and PLM indices were significantly higher in GBS patients with *p* value < 0.0001 for each parameter (Table 1).

One month after immunotherapy, the GBS patients revealed significant improvements in the ODSS and NPS with *p* value 0.0001 and 0.001 respectively when compared with initial assessment. The HAS and MADRS showed non-significant changes with *p* value 0.22 and 0.398 respectively. The study also showed that motor deficits measured by the ODSS were not correlated with the patients’ age, HAS, MADRS, mean sleep latencies of the MSLT, and other examined PSG parameters (Table 3). At the same time, the ODSS improvement was 1.8 ± 0.8 which was not correlated with the degree of HAS and MADRS improvements (3.05 ± 1.36 and 3.1 ± 3.5 with *p* values 0.303 and 0.638 respectively).

**Table 3:** Correlations of the Overall Disability Sum Score and other studied parameters, 1 month after finishing immunotherapy

	Overall Disability Sum Score after 1 month	
	*r*	*p*
Patients’ ages	0.13	0.56
Neuropathic Pain Scale	0.05	0.81
Hamilton Anxiety Scale	− 0.27	0.24
Montgomery–Åsberg Depression Rating Scale	− 0.11	0.62
Mean sleep latency of MSLT (min)	− 0.10	0.66
Total sleep time (hours)	− 0.02	0.92
Wake after sleep onset (hours)	− 0.07	0.75
Sleep efficiency (%)	0.08	0.72
Sleep stage transition index (/hour)	0.024	0.30
Apnea hypopnea index (/hour)	− 0.15	0.52
Arousal index (/hour)	0.07	0.76
Snore Index (/hour)	0.09	0.69
Periodic limb movements index (/hour)	− 0.013	0.95

*MSLT* multiple sleep latency test

Regarding sleep architecture, GBS patients showed non-significant changes in each of the mean latencies of MSLT, SSTI, AHI, DI, AI, and PLM indices. Each of TST, WASO, SE, lowest O_2_ saturation, and SI showed mild significant improvement with *p* value 0.034, 0.014, 0.01, 0.042, and 0.034 respectively.

## Discussion

Guillain Barré Syndrome is an acute monophasic post-infectious immune-mediated peripheral neuropathy with sensory disturbances, motor weakness, and diminished or absent reflexes with or without cranial nerve affection. Patients with GBS usually need prolonged hospital stay and often experience anxiety and sleep disturbances (Karkare et al. 2013).

This work studied the possible psychiatric and sleep abnormalities in GBS patients. The study showed that GBS is more common in middle-aged males and about half of the cases had cranial nerve affection, and neurophysiology showed that most of the patients were predominantly demyelinating motor neuropathy. The improvements in the sensori-motor manifestations were higher among those lacking cranial nerve affection, milder disabilities, demyelinating neuropathies, younger ages, and lower CSF proteins. These results are in accordance with the work of González-Suárez et al. (2013) and Fokke et al. (2014) who studied the predictors of outcome in GBS patients and concluded that bad prognosis is demonstrated among older-aged GBS patients, severe motor deficits at onset, injured cranial nerves, and axonal lesion patterns in the neurophysiological examinations.

The study declared that after exclusion of cases with severe motor disability, GBS patients experienced moderate–severe anxiety and mild-moderate depressive symptoms during the early phases of the disease before immunotherapy which were positively correlated with the degree of motor disability. Both anxiety and depressive symptoms were non-significantly correlated with the rapidity of symptoms progression measured by the duration between symptoms onsets and admission times which are possibly explained by the exclusion of GBS patients with severe motor disabilities that may exhibit more rapid disease progression. These results are passing with the work of Bernsen et al. (2010) who found a high rate of anxiety among GBS patients in early disease stages and attribute this to ICU admission, fear of need of assisted ventilation and haziness in the expectation about the future prognosis, but on the contrary, they found positive correlation with rapidity of disabilities possibly due to non-exclusion of those needed assisted ventilation who may have more rapid disease course. Brousseau et al. (2005) went beyond these results, and they found higher rate of anxiety, depressive symptoms, and brief reactive psychosis in GBS patients than that in ICU-admitted patients for other indications with near degree of disability and supposed that psychiatric symptoms are parts of the clinical manifestations of GBS.

One month after immunotherapy, the patients showed good recovery of the sensori-motor manifestations while the anxiety and depressive symptoms showed non-parallel improvement which means that they need longer time, may not resolve spontaneously, and are in need of specific psychiatric treatment. These results agree with Bernsen et al. (2010) who found psychological dysfunctions in GBS patients persisting up to 1 year from the disease onset. Brousseau et al. (2005) advise the use of modest doses of specific serotonin reuptake inhibitors, anticonvulsants, and supportive psychotherapy to ameliorate the symptoms of anxiety and depression during rehabilitation of GBS patients for better prognosis and earlier independence.

Regarding the sleep assessment, the study showed EDS in initial evaluation of GBS patients as evidenced by shortened mean latencies of MSLT. This result agrees with that of Gao et al. (2016) who found EDS in GBS patients through using the Pittsburgh Sleep Quality Index. Polysomnographic assessment during this stage showed marked prolongation of WASO on the expense of reduction in TST, decreased SE, and increased SSTI. Sleep architecture showed shortened N2% and REM% of TST with nearly normal N1% and relatively prolonged N3% of TST. The PSG abnormalities and EDS were proportional with the severity of anxiety symptoms. These results are partly going with the work of Cochen et al. (2005) who found shortening and fragmentation of all sleep stages in GBS patients.

The study also showed increased AHI, DI, AI, and SI with shortening of the PTT pointing to the presence of mild–moderate obstructive sleep apnea (OSA) syndrome in the studied patients especially those who had cranial nerve affection and bulbar symptoms. The OSA was mild–moderate in severity (AHI was 19.11 ± 7.75) possibly due to exclusion of patients with severe motor deficit. These results agree with that of Chokroverty (2003) who stated that chest wall and respiratory muscle fatigue with extension of the REM sleep hypotonia to affect the respiratory muscles (the extra-ocular and respiratory muscles are normally not involved in the REM sleep hypotonia) may be the cause of OSA in GBS patients. Periodic limb movement index also showed significant increase in early disease stages, and this result is also passing with the work of Gao et al. (2016).

Re-evaluation of the patients 1 month after immunotherapy showed mild improvement of MSLT and some PSG parameters but still significantly impaired when compared with the control group which point to that sleep disturbances in GBS patients may take long time to improve and may need treatment either pharmacologically or by continuous positive airway pressure ventilation (CPAP) to reduce the patient’s suffering. This observation is in accordance with that of Cochen et al. (2005) and Chokroverty (2003) who declared that GBS patients exhibit a lot of sleep abnormalities including REM behavior disorders, transitory hypocretin-1 transmission decrease, and sleep-disordered breathing which may need management for earlier patients’ recovery and independence.

### Limitations

The limitations of this study are the small sample size and the need for longer follow-up period which is intended to be overcame in a second phase of the study. Also, exclusion of GBS patients with impending respiratory failure is added to the study limitations.

## Conclusion

Sleep and psychiatric disturbances are common manifestations of GBS which are not proportional with the degree of motor disability and rapidity of progression. These disturbances need special attention in the management protocol for earlier patients' independence and better functional outcome.

## Abbreviations

AHI: Apnea hypopnea index
AI: Arousal index
BMI: Body mass index
DI: Desaturation index
GBS: Guillain Barré Syndrome
HAS: Hamilton Anxiety Scale
MADRS: Montgomery–Åsberg Depression Rating Scale
MSLT: Multiple sleep latency test
N2: Non-rapid eye movement sleep stage 2
NPS: Neuropathy pain scale
ODSS: Overall disability sum score
PLMI: Periodic limb movement index
PSG: Polysomnogram
PTT: Pulse transit time
REM: Rapid eye movement
SE: Sleep efficiency
SI: Snore index
SSTI: Sleep stage transition index
TIB: Total in bed time
TST: Total sleep time
WASO: Wake after sleep onset
